# Defining new clinically derived criteria for high disease activity in non-systemic juvenile idiopathic arthritis: a Finnish multicentre study

**DOI:** 10.1093/rap/rky044

**Published:** 2018-10-24

**Authors:** Maria Backström, Pirjo Tynjälä, Kristiina Aalto, Heikki Ylijoki, Anne Putto-Laurila, Minna-Maija Grönlund, Johanna Kärki, Paula Keskitalo, Sirja Sard, Heini Pohjankoski, Maiju Hietanen, Silke Witter, Helena Lehto, Eliisa Löyttyniemi, Paula Vähäsalo

**Affiliations:** 1 Department of Pediatrics, Vaasa Central Hospital, Vaasa, Finland; 2 Department of Pediatrics, South-Karelian Central Hospital, Lappeenranta, Finland; 3 Poison Information Center, Helsinki University Central Hospital, Helsinki, Finland; 4 Paediatric Department, Children’s Hospital, Helsinki University Central Hospital, University of Helsinki, Helsinki, Finland; 5 Department of Pediatrics, Satakunta Central Hospital, Pori, Finland; 6 Department of Pediatrics, Turku University Hospital, Turku, Finland; 7 Department of Pediatrics, Kanta-Häme Central Hospital, Hämeenlinna, Finland; 8 Department of Children and Adolescents, Medical Research Center Oulu, Oulu University Hospital and PEDEGO Research Unit, University of Oulu, Oulu, Finland; 9 Department of Pediatrics, Päijät-Häme Central Hospital, Lahti, Finland; 10 Department of Pediatrics, Central Finland Central Hospital, Jyväskylä, Finland; 11 Department of Biostatistics, University of Turku, Turku, Finland

**Keywords:** juvenile idiopathic arthritis, disease activity, outcome assessment

## Abstract

**Objectives:**

To redefine criteria for high disease activity (HDA) in JIA, to establish HDA cut-off values for the 10-joint Juvenile Arthritis Disease Activity Score (JADAS10) and clinical JADAS10 (cJADAS10) and to describe the distribution of patients’ disease activity levels based on the JADAS cut-off values in the literature.

**Methods:**

Data on 305 treatment-naïve JIA patients were collected from nine paediatric units treating JIA. The median parameters of the JADAS were proposed to be the clinical criteria for HDA. The cut-off values were assessed by using two receiver operating characteristics curve–based methods. The patients were divided into disease activity levels based on currently used JADAS cut-off values.

**Results:**

We proposed new criteria for HDA. At least three of the following criteria must be satisfied in both disease courses: in oligoarthritis, two or more active joints, ESR above normal, physician global assessment (PGA) of disease activity ≥2 and parent/patient global assessment (PtGA) of well-being ≥2; in polyarthritis, six or more active joints, ESR above normal, PGA of overall disease activity ≥4 and PtGA of well-being ≥2. The HDA cut-off values for JADAS10 (cJADAS) were ≥6.7 (6.7) for oligoarticular and ≥15.3 (14.1) for polyarticular disease. The distribution of the disease activity levels based on the JADAS cut-off values in the literature varied markedly based on which cut-offs were used.

**Conclusion:**

New clinically derived criteria for HDA in JIA and both JADAS and cJADAS cut-off values for HDA were proposed.

**Key messages**

- New clinically derived criteria for HDA in JIA were proposed.
- JADAS10 and cJADAS cut-off values for HDA were suggested to be ≥6.7 in oligoarthritis.
- The corresponding values were suggested to be ≥15.3 and ≥14.1 in polyarthritis.

## Introduction

JIA is a heterogeneous group of chronic arthritides in childhood with variable presentations, treatments and outcomes [1]. JIA is diagnosed in patients <16 years of age when synovial inflammation lasts for >6 weeks and its aetiology is unknown. JIA includes seven categories, revised by the ILAR paediatric task force [2]. Chronic synovial inflammation in JIA can lead to long-term consequences, such as destruction of joints, reduced growth, osteoporosis, chronic pain and visual impairment [1]. The goal of the medical treatment is clinically inactive disease (CID), which can usually be achieved through early aggressive treatment [3–5].

Treatment decisions are guided by the continuous and systematic evaluation of disease activity. Thus objective disease activity measures and definitions for disease activity levels are needed to enable the comparison of outcomes in clinical research and follow-up. The Juvenile Arthritis Disease Activity Score (JADAS), especially the clinical JADAS (cJADAS, excluding ESR), has been developed to measure disease activity in JIA [6, 7]. JADAS is the paediatric version of the adult DAS [8] and consists of four parameters: active joint count (AJC), physician global assessment (PGA) of disease activity, parent’s evaluation of the child’s overall well-being [i.e. parent/patient global assessment (PtGA) of well-being] and ESR. The JADAS can be determined for 71, 27 or 10 active joints (JADAS71, JADAS27 or JADAS10, respectively).

Several attempts have been made to divide the disease activity into various levels based on clinical criteria or expert opinion [9–14] (Table 1). Moreover, a few suggestions for establishing JADAS and cJADAS cut-off values for these disease activity levels have been reported [14, 16–18] (Table 2).

**Table 1:** Different definitions of disease activity levels in JIA

Disease activity	Oligoarticular disease	Polyarticular disease
	Magni-Manzoni *et al*. 2008 [11]	
Minimal disease activity	PGA of disease activity ≤2.5 of 10	PGA of disease activity ≤3.4 of 10
	Number of swollen joints 0	PtGA of well-being ≤2.1 of 10
		Number of swollen joints ≤1
	Beukelman *et al*. 2011 [12]	
	The following criteria must be satisfied:	The following criteria must be satisfied:
Low disease activity	≤1 active joints	≤4 active joints
	ESR or CRP level normal	ESR or CRP level normal
	PGA of disease activity <3 of 10	PGA of disease activity <4 of 10
	PtGA of well-being <2 of 10	PtGA of well-being <2 of 10
Moderate disease activity	One or more features of the LDA level and fewer than three features of HDA	One or more features of the LDA level and fewer than three features of HDA
High disease activity	Three of the following criteria must be satisfied:	Three of the following criteria must be satisfied:
	≥2 active joints	≥8 active joints
	ESR or CRP level greater than twice the upper limit of normal	ESR or CRP level greater than twice the upper limit of normal
	PGA of disease activity ≥7 of 10	PGA of disease activity ≥7 of 10
	PtGA of well-being ≥4 of 10	PtGA of well-being ≥5 of 10
	Bulatović Ćalasan *et al*. 2014 [13]	
Low disease activity	No medication or NSAID as monotherapy	No medication or NSAID as monotherapy
	or	or
	Stopping MTX or biologic medication (not due to adverse effects)	Stopping MTX or biologic medication
High disease activity	Starting MTX, oral corticosteroid or biologic medication	Starting MTX, oral corticosteroid or biologic medication
	Consolaro *et al*. 2014 [14]	
High disease activity	Starting a DMARD or bDMARD or	Starting a DMARD, systemic corticosteroid therapy or bDMARD or
	Intra-articular corticosteroid administration in ≥1 joint	Intra-articular corticosteroid administration in ≥3 joints

bDMARD: biologic DMARD.

**Table 2:** JADAS10 and cJADAS10 cut-off values according to Backström *et al.* [15] and Consolaro *et al*. [14–18] for select disease activity levels of JIA, CID, LDA, MDA and HDA

Disease activity level	Selected cut-offs		Disease activity levels used as reference
	Oligoartricular disease	Polyarticular disease	
CID		
JADAS10	0.5 [15]	0.7 [15]	Wallace *et al*. [9]
	1.0 [17]	1.0 [17]	Wallace *et al*. [9]
	1.5 [16]	2.6 [16]	Experts subjective opinion [16]
cJADAS10	0.5 [15]	0.7 [15]	Wallace *et al*. [9]
	1.0 [18]	1.0 [18]	Wallace *et al*. [9]
	1.2 [16]	2.4 [16]	Experts subjective opinion [16]
LDA			
JADAS10	0.6–2.7 [15]	0.8–3.9 [15]	Beukelman *et al*. [12]
	1.1–2.0 [17]	1.1–3.8 [17]	Magni-Manzoni *et al*. [11]
	1.6–3.9 [16]	2.7–5.1 [16]	Experts subjective opinion [16]
cJADAS10	0.6–2.7 [15]	0.8–3.9 [15]	Beukelman *et al*. [12]
	1.1–1.5 [18]	1.1–2.5 [18]	Magni-Manzoni *et al*. [11]
	1.3–3.4 [16]	2.5–5.1 [16]	Experts subjective opinion [16]
MDA			
JADAS10	≥2.8 [15]	≥4.0 [15]	Beukelman *et al*. [12]
	2.1–4.2 [14, 17]	3.9–10.5 [14, 17]	Magni-Manzoni *et al*. [11], Consolaro *et al*.[14]
	4.0–16.4 [16]	5.2–18.9 [16]	Experts subjective opinion [16]
cJADAS10	≥2.8 [15]	≥4.0 [15]	Beukelman *et al*. [12]
	1.6–4.0 [14, 18]	2.6–8.5 [14, 18]	Magni-Manzoni *et al*. [11], Consolaro *et al*. [14]
	3.5–14.3 [16]	5.2–19.0 [16]	Experts subjective opinion [16]
HDA			
JADAS10	>4.2 [14]	>10.5 [14]	Consolaro *et al*. [14]
	>16.4 [16]	>18.9 [16]	Experts subjective opinion [16]
cJADAS10	>4.0 [18]	>8.5 [18]	Consolaro *et al*. [14]
	>14.3 [16]	>19.0 [16]	Experts subjective opinion [16]

**Table 3:** Clinical characteristics in a Finnish cohort of 305 patients with newly diagnosed yet untreated non-systemic JIA with a complete dataset and 208 patients with an incomplete dataset

Characteristics	Oligoartricular disease		Polyartricular disease	
	Complete dataset	Incomplete dataset	Complete dataset	Incomplete dataset
Patients, *n* (%)	152 (54.3)	128 (45.7)	153 (65.7)	80 (34.3)
Females, *n* (%)	108 (71.1)	88 (68.8)	108 (70.6)	57 (71.3)
Age, years, median (range)	7.33 (1.42–16.05)	7.65 (1.08–16.84)	8.22 (0.75–16.25)	6.02 (1.25–16.28)
Subcategories of JIA				
Oligoarthritis, persisted M08.4, *n* (%)	121 (79.8)	98 (76.6)	0 (0.0)	0 (0.0)
Oligoarthritis, extended M08.4, *n* (%)	0 (0.0)	0 (0.0)	9 (4.1)	13 (16.3)
Polyarthritis, RF-negative M08.3, *n* (%)	0 (0.0)	0 (0.0)	116 (75.8)	60 (75.1)
Polyarthritis, RF-positive M08.0, *n* (%)	3 (2.0)	1 (0.8)	16 (10.5)	2 (2.5)
Enthesitis-related arthritis M08.1, *n* (%)	19 (12.5)	14 (10.9)	5 (3.3)	0 (0.0)
Psoriatic arthritis M09*L40.5, *n* (%)	3 (2.0)	4 (3.1)	4 (2.6)	2 (2.5)
Other juvenile arthritis M08.8, *n* (%)	1 (0.7)	1 (0.8)	1 (0.7)	1 (1.3)
Undifferentiated arthritis M08.9, *n* (%)	5 (3.3)	10 (7.8)	2 (1.3)	2 (2.5)
PGA, cm, median (IQR)	1.85 (1.0–2.5)^a^	2.0 (1.0–2.3)^b^	3.5 (2.45–4.5)^f^	3.4 (1.9–5.0)^g^
PtGA of well-being, cm, median (IQR)	1.5 (0.5–3.0)^a^	4.0 (1.5–8.75)^c^	2.2 (1.0–4.75)^f^	0.6 (0–0.6)^h^
Joints with active arthritis, *n*, median (IQR)	2 (1–2)^a^	2 (1–2)^d^	6 (4–10)^f^	5 (4–8)^i^
ESR, mm/h, median (IQR)	9.0 (5.0–18.0)^a^,*	7.0 (4.0–16.0)^e,^*	15.0 (7.5–30.5)^f,^**	9.0 (5.0–18.8)^j,^**

a *n* = 152.

b *n* = 49.

c *n* = 4.

d *n* = 128.

e *n* = 126.

f *n* = 153.

g *n* = 34.

h *n* = 2.

i *n* = 78.

j *n* = 76.

* *P* = 0.037, **P = 0.019.

**Table 4:** A new proposal for the clinical criteria of HDA in JIA

High disease activity (at least three of the criteria below must be satisfied)
Oligoarthritis
Two or more active joints
ESR above normal
PGA of disease activity ≥2
PtGA of well-being ≥2
Polyarthritis
Six or more active joints
ESR above normal
PGA of overall disease activity ≥4
PtGA of well-being ≥2

**Table 5:** Optimal JADAS10 cut-off values

	Closest to point cut-off value (sensitivity/specificity)	Youden index cut-off value (sensitivity/specificity)	AUC	Selected cut-offs	Correct classification rate, %	Too low/too high, %
Oligoarticular disease						
High disease activity						
JADAS10	6.6 (0.89/0.72)	6.6 (0.89/0.72)	0.87	6.6	86.4	13.6/0
cJADAS10	6.6 (0.89/0.80)	6.6 (0.89/0.80)	0.88	6.6	86.4	13.6/0
Polyarticular disease						
High disease activity						
JADAS10	15.2 (0.86/0.91)	15.2 (0.86/0.91)	0.93	15.2	84.5	15.5/0
cJADAS10	14.0 (0.90/0.78)	14.0 (0.90/0.78)	0.92	14.0	82.8	17.2/0

The cut-off values are determined by two different receiver operating characteristics curve–based methods, the one closest to point (0, 1) and the Youden index [21], for HDA as proposed in the present study. AUC: area under the curve.

The Wallace preliminary definition of CID [9] and the American College of Rheumatology provisional criteria for CID [10] have been used quite uniformly by paediatric rheumatologists. However, it was recently shown that when CID is defined by Wallace’s preliminary criteria and the JADAS cut-off for CID, less than half of the patients had CID based on both definitions [19]. Several definitions for minimal or low disease activity (LDA), moderate disease activity (MDA) and high disease activity (HDA) exist [11–14]. In a recent study we showed that considerable overlap exists among the various definitions [20]. Approximately 20% of patients with LDA according to Beukelman *et al*. [12] had HDA according to Consolaro *et al*. [14, 20]. Hence the current classification of disease activity, especially the criteria for HDA, seem to be heterogeneous and incongruous. An objective, uniformly used classification of disease activity is needed to enable benchmarking for clinical services and better comparison of reported outcomes in research. The objective of the current study was to define new and clinically derived criteria for HDA and to establish HDA cut-off values for JADAS10 and cJADAS10. We suggest that a treatment-naïve patient with average or active JIA can be considered to represent a patient with HDA. Another objective was to describe the distribution of disease activity levels based on the JADAS cut-off values in the literature in our cohort of newly diagnosed treatment-naïve JIA patients.

## Methods

To define new clinical criteria for HDA, we retrospectively collected data between January 2013 and February 2016 on the first visit of all recently diagnosed, consecutive DMARD-naïve patients with non-systemic JIA. Patients with systemic JIA were excluded. Collection of the data took place in nine Finnish hospitals with paediatric rheumatology outpatient departments. Both secondary and tertiary centres were included. Data on age, gender, ILAR category of JIA [2], AJC, ESR, PGA of disease activity using a 10-cm linear visual analogue scale (VAS), PtGA of well-being using a 10-cm linear VAS and RF levels were obtained. On reporting, we preferred JADAS10 scores, because JADAS27 excluding clinically significant joints (e.g. midtarsal and TMJs) and JADAS71 were too time consuming. Patients were categorized into each disease activity level based on the JADAS cut-off values in the literature [15–18].

### Statistics

To assess clinical criteria for HDA, we used the median and interquartile range (IQR) as descriptive statistics and JADAS core set values, i.e. AJC, ESR, PGA of disease activity and PtGA of well-being. Due to the skewed distribution of parameters, non-parametric Spearman’s ρ correlation coefficient and Mann–Whitney U test were used to assess groups and differences among them with regard to continuous variables and the chi-squared test was used to assess nominal parameters. We determined the cut-off values for JADAS10 and cJADAS10 using two receiver operating characteristics curve–based methods: the one closest to point 0.1 and the Youden index [21], which yielded the highest degree of combined sensitivity and specificity. The analyses were performed with SPSS Statistics, version 20 (IBM, Armonk, NY, USA) and the SAS System for Windows, version 9.4 (SAS Institute, Cary, NC, USA).

### Ethics

The study complies with the Declaration of Helsinki. The retrospective data were gathered without patient identification. Based on Finnish ethical regulations, no patient consent or ethical committee approval was needed. Instead, permission was obtained from the directors of the hospitals participating in this study.

## Results

In defining the clinical criteria for HDA, data on 513 consecutive recently diagnosed DMARD-naïve patients with non-systemic JIA were explored. The data on 208 patients were incomplete. The median age of the 305 patients with complete data was 7.8 years (range 0.8–16.3) and 216 of them (70.8%) were girls (Table 3). Seven patients (2.3%) were classified as CID, 40 (13.1%) as LDA, 246 (80.6%) as MDA and 12 (3.9%) as HDA based on the disease activity criteria of Beukelman *et al*. [12]. The median JADAS10 was 5.4 (IQR 3.5–8.0) in oligoarticular patients and 13.4 (IQR 9.1–17.7) in polyarticular patients. The median of the treatment-naïve patients’ JADAS was used in defining HDA (Table 4). When the HDA criteria were applied to the original cohort, 102 (33.4%) patients were classified as having HDA. In those with oligoarticular disease, an optimal HDA cut-off value for both JADAS10 and cJADAS10 was ≥6.7, and in polyartricular disease it was ≥15.3 and ≥14.1, respectively (Table 5).

**Fig. 1:**
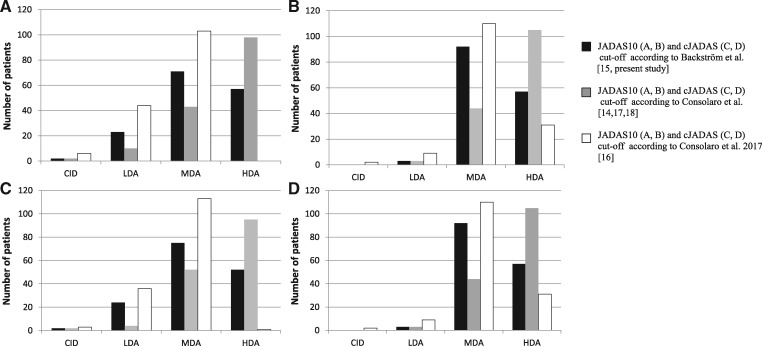
Distribution of disease activity levels in 305 treatment-naïve oligoartricular (**A**, **C**) and polyartricular (**B**, **D**) patients.

We divided patients in our cohort of recent-onset treatment-naïve JIA into disease activity levels based on JADAS cut-off values according to Backström *et al.* [15, present study], Consolaro *et al*. [14, 17] and Consolaro *et al*. [16] and cJADAS cut-off values according to Consolaro *et al*. [18] (Fig. 1). When the recently published cut-off values by Consolaro *et al*. [16] were used, none of those with oligoarticular disease had HDA and altogether eight of them had CID. Of the eight patients with CID, five had an inflamed joint. When cut-off values proposed by Backström *et al*. [15] were applied, two patients had CID and the AJC was zero in both of these patients.

In the entire cohort of 305 patients with complete data the median AJC was 3 (IQR 1–6), the physician’s VAS was 2.5 (IQR 1.5–3.9) and the patient/parent VAS was 2.0 (IQR 0.9–4.0). The correlation was weak both between the patient/parent VAS and the AJC (*r*_s_ = 0.25, *P* < 0.001) and between the patient’s and physician’s VAS (*r*_s_ = 0.37, *P* < 0.001). The correlation was strong between the physician’s VAS and the AJC (*r*_s_ = 0.72, *P* < 0.001).

A total of 208 patients with missing JADAS values had slightly lower AJCs (median 2 *vs* 3; *P* = 0.011) and ESRs (7 *vs* 12; *P* < 0.001) compared with those with complete data. These two groups were comparable in age, physician’s VAS and patient/parent VAS (Table 3). Fifty percent of those with complete and 62% of those with incomplete data had an oligoarticular disease course. When we divided our cohort into patients with oligoarticular and polyarticular disease, the two groups with complete and incomplete data were comparable in age, AJC, physician’s VAS and patient/parent VAS. ESR was slightly lower in the patients with missing JADAS values (Table 3).

## Discussion

In this study we redefined the clinical criteria for HDA based on the median JADAS core set criteria in a cohort of recent-onset treatment-naïve non-systemic JIA patients. The data for the proposed criteria were derived from a consecutive, national cohort of real-life patients with recently diagnosed, DMARD-naïve JIA. We clearly showed earlier that the existing definitions of HDA [12–14] are inconsistent [20], and because they are consensus based and not population based, we suggest that a treatment-naïve patient with average or more active JIA can be considered to represent a patient with HDA.

In our study, the optimal HDA cut-off value for JADAS10 was ≥6.7 for oligoarticular and ≥15.3 for polyarticular disease. These values were somewhat higher than in an earlier work by Consolaro *et al*. [14], in which 4.2 was used for oligoarticular disease and 10.5 was used for polyarticular disease. This is reasonable because, by definition, patients with low JADAS scores can still have HDA according to Consolaro *et al*. [14]. By their definition, injection of cortisone into one active joint classifies the patient as HDA if the patient has oligoarticular disease. Regarding the use of intra-articular corticosteroids, various country codes exist depending on national guidelines and the accessibility of injections. In Finland, the most highly active joints are usually given steroid injections rather than systemic steroids being prescribed. Thus in a Finnish JIA cohort, the proportion of patients with HDA, based on previous recommendations [14], is high [20]. On the other hand, Beukelman’s criteria for HDA [12] were set so high that the patients fulfilling them were hard to find even in our cohort with recent-onset treatment-naïve JIA.

Of interest is a recent study where only 2% of the 49 young adults with polyarticular JIA in the study fulfilled HDA criteria with the 28-joint DAS (DAS28) cut-off values but up to 27% fulfilled the HDA criteria based on JADAS10 [22]. A reason for this discrepancy is that the AJC with JADAS10 is higher compared with the DAS28, as the DAS28 does not include all joints. Another explanation may be that the currently used clinical criteria for HDA on which the JADAS10 HDA cut-off relies has been set too low [14]. In the future, it would be interesting to investigate how the HDA criteria and cut-off values proposed in this study correlate with adult-based disease outcome parameters.

In the present study, 208/513 patients had incomplete datasets. This is not uncommon in clinical datasets but raises the concern that the final dataset in our study may not be reflective of all newly diagnosed patients. Polyartricular disease was slightly less common in those with incomplete data. Because the definition for HDA is different with oligoarticular and polyarticular disease, the reliability of the results of our study should not be markedly undermined by the slight difference in disease pattern. Moreover, in a recent study from the UK, the activity levels of 651 oligoarticular and 280 polyarticular recent-onset DMARD-naïve patients were in line with our cohort’s activity level [23]. However, we cannot be definitive that these clinically derived HDA criteria are relevant outside of our cohort. The relevance needs to be verified in a larger, preferably multinational cohort.

Recently, in 2017, Consolaro *et al*. [16] presented new JADAS and cJADAS cut-off values where disease activity levels determined by expert opinions served as a reference. These cut-off values were higher than the ones in previous studies [14, 15, 17, 18]. The present study revealed that with the use of these cut-off levels there will be some patients with active joints classified as having CID; five patients had one active joint and they were simultaneously classified as CID based on the cut-off values of Consolaro *et al*. 2017 [16]. We prefer cut-off values that are low enough to exclude those with active joints from being classified as CID. When using the JADAS cut-off values for HDA from 2017 [16], none of the oligoarticular patients in our cohort of recent-onset DMARD-naïve patients was classified as HDA, which seems to indicate these cut-off values are too high, even regarding HDA.

The HDA cut-off values in the present study still require further validation using the same objective disease activity levels used in our study.

In conclusion, we defined the new clinical criteria for HDA as follows: for oligoarthritis, two or more active joints, ESR above normal, PGA of disease activity ≥2, PtGA of well-being ≥2 and at least three of these must be satisfied; for polyarthritis, six or more active joints, ESR above normal, PGA of overall disease activity ≥4, PtGA of well-being ≥2 and at least three of these must be satisfied. The HDA cut-off values for JADAS10 (cJADAS) were ≥6.7 (6.7) for oligoarticular and ≥15.3 (14.1) for polyarticular disease. In the future, it will be important to discuss and establish an international consensus on the clinical criteria for disease activity levels to be able to determine and validate robust JADAS cut-off values. These required cut-off values of JADAS are needed to enable better description and valid comparison of various patient cohorts in research and day-to-day clinical practice.

## Funding

This work was supported by state funding for university-level health research, Vaasa Central Hospital, Finland; the Finnish Pediatric Research Foundation; the Alma and K. A. Snellman Foundation, Oulu, Finland and the Päivikki and Sakari Sohlberg Foundation, Finland.

*Disclosure statement*: The authors have declared no conflicts of interest.
